# SNP and Haplotype Interaction Models Reveal Association of Surfactant Protein Gene Polymorphisms With Hypersensitivity Pneumonitis of Mexican Population

**DOI:** 10.3389/fmed.2020.588404

**Published:** 2021-01-05

**Authors:** Chintan K. Gandhi, Chixiang Chen, Shaili Amatya, Lili Yang, Chenqi Fu, Shouhao Zhou, Rongling Wu, Ivette Buendía-Roldan, Moisés Selman, Annie Pardo, Joanna Floros

**Affiliations:** ^1^Center for Host Defense, Inflammation, and Lung Disease (CHILD) Research, Department of Pediatrics, Pennsylvania State University College of Medicine, Hershey, PA, United States; ^2^Department of Public Health Science, Pennsylvania State University College of Medicine, Hershey, PA, United States; ^3^School of First Clinical Medicine, Nanjing University of Chinese Medicine, Nanjing, China; ^4^Unidad de Investigación, Instituto Nacional de Enfermedades Respiratorias “Ismael Cosio Villegas”, Mexico City, Mexico; ^5^Universidad Nacional Autonoma de Mexico, Mexico City, Mexico; ^6^Department of Obstetrics & Gynecology, Pennsylvania State University College of Medicine, Hershey, PA, United States

**Keywords:** SNP-SNP interaction, surfactant protein gene polymorphism, *SFTPA1*, *SFTPA2*, *SFTPB*, *SFTPC*, *SFTPD*, genetic susceptibility

## Abstract

**Background:** Hypersensitivity pneumonitis (HP) is an interstitial lung disease caused by inhalation of common environmental organic particles. Surfactant proteins (SPs) play a role in innate immunity and surfactant function. We hypothesized that single nucleotide polymorphisms (SNPs) or haplotypes of the SP genes associate with HP.

**Methods:** Seventy-five HP patients caused by avian antigen and 258 controls, asymptomatic antigen exposed and non-exposed were enrolled. SNP association was performed using logistic regression analysis and SNP-SNP interaction models.

**Results:** Based on odds ratio, regression analyses showed association of (a) rs7316_G, 1A^3^ (protective) compared to antigen exposed; (b) male sex, smoking, rs721917_T and rs1130866_T (protective) compared to non-exposed controls with HP; (c) compared to antigen exposed, 25 interactions associated with HP in a three-SNP model; (d) compared to non-exposed, (i) rs1136451 associated with increased, whereas rs1136450 and rs1130866 associated with lower HP risk, (ii) 97 interactions associated with HP in a three-SNP model. The majority of SNP-SNP interactions associated with increased HP risk involved SNPs of the hydrophilic SPs, whereas, the majority of interactions associated with lower HP risk involved SNPs of both hydrophilic and hydrophobic SPs; (e) haplotypes of SP genes associated with HP risk.

**Conclusions:** The complexity of SNPs interactions of the *SFTP* genes observed indicate that the lung inflammatory response to avian antigens is modulated by a complex gene interplay rather than by single SNPs.

## Introduction

Hypersensitivity pneumonitis (HP) is an interstitial lung disease caused by an abnormal immune response to a wide variety of inhaled environmental antigens, mainly small organic particles (<5 μm) that reach the alveoli (1). These antigens provoke an exaggerated immune response in susceptible individuals (1, 2). HP is seen worldwide, and the most common implicated antigens are actinomyces species, fungi, and bird proteins (1). The pigeon breeder's disease caused by proteins from avian serum, feces, and feathers is the most common form of HP in Mexico (3). Given the universal and wide distribution of the offending antigens, it is unclear why only few individuals develop the disease, indicating the complex interaction between environment and genetic factors. Nonetheless, the host genetic factors that may play a role in HP susceptibility are understudied (1, 2).

Although pulmonary surfactant or its components have the potential to be contributors to the pathogenesis of HP, very little to no work has been done in this regard. Pulmonary surfactant is a complex mixture of 90% lipids and 10% proteins. The surfactant specific proteins are SP-A1, SP-A2, SP-B, and SP-C. Though SP-D is not part of the functional surfactant complex, it is grouped with the other surfactant proteins because it coisolates with them. SP-B and SP-C are hydrophobic proteins and play a role primarily in lowering the surface tension and stabilizing alveoli (4), whereas, SP-A and SP-D are hydrophilic proteins and play a role in innate immunity and host defense (5, 6), although SP-A also contributes to surfactant-related function (6). The human SP-A locus includes two functional genes, *SFTPA1* and *SFTPA2*, in opposite transcriptional orientation (7), encoding SP-A1 and SP-A2, respectively. Several genetic polymorphisms for each *SFTPA* gene are found frequently in the general population (8). SP-B, SP-C, and SP-D are each encoded by a single gene, *SFTPB, SFTPC*, and *SFTPD*, respectively (9), and several polymorphisms have been described for each (10–12).

Single nucleotide polymorphisms (SNPs) of the SP genes have been shown to associate with various acute and chronic pulmonary diseases, such as idiopathic pulmonary fibrosis (IPF) (13), chronic obstructive pulmonary disease (COPD) (14, 15), acute respiratory distress syndrome (10), cystic fibrosis (16), and neonatal respiratory distress syndrome (RDS) (17–20). More importantly, we have shown an association of SP gene polymorphisms with IPF (13), COPD (14), and tuberculosis (21) in the Mexican population. Furthermore, some of the common SP-A variants have been shown to differentially affect function (22, 23) and regulation (24–27) of alveolar macrophages in an animal model, as well as SP-A variants have been associated with differential lung function mechanics and survival of mice (28–30). For the current study, we selected from physiologically and biologically relevant genes, 17 well-characterized SP SNPs that have been shown to associate with various acute and chronic pulmonary diseases (12, 31). Previous studies have shown increased SP-A in bronchoalveolar lavage (BAL) and alveolar macrophages, and elevated serum SP-A and SP-D concentration in HP patients compared to controls (32–34). Though the exact mechanisms are unknown, HP pathogenesis may include alveolar epithelial injury by altered immune response and increased leakage of SP-A and SP-D from the alveolar to the vascular compartment. Moreover, previous research showed altered concentration of SP-A, SP-B, and phospholipids in IPF, HP, and sarcoidosis patients (34, 35). Taken together, it is likely that SPs play a critical role in HP.

HP is characterized by an abnormal immune response leading to chronic inflammation and abnormal lung function and is probably the result of complex interactions of genetic and environmental factors. Due to the importance of SPs in normal lung function, innate immunity and host defense of the lung, we hypothesized that natural genetic variants of SPs and their interactions are associated with HP in Mexican population.

## Subjects and Methods

### Study Population

The study was conducted at the National Institute of Respiratory Diseases (INER) in Mexico City and the protocol was approved by the Ethics Committee. INER is a tertiary referral and one of the National Institutes of Health centers in Mexico. The study cohort compromised 3 groups (age > 18 years) and at least 3 generations of study participants were born in Mexico.

Group 1 (Cases) was composed of 75 consecutive unrelated patients 18 years of age or older with a diagnosis of HP (Table 1). HP was diagnosed as previously described using clinical characteristics, history of antecedent antigen exposure and specific antibodies against avian proteins, pulmonary function tests, high-resolution computed tomography, BAL findings (all 75 patients), and/or surgical biopsy when available (23 out of 75 patients) (1, 36). Attending clinicians systematically recorded the relevant data in every patient before making the final diagnosis and was confirmed by a multidisciplinary team. As per recently published official clinical practice guidelines from American Thoracic Society, all enrolled patients in the current study were classified to have a fibrotic HP (mixed inflammatory plus fibrosis) (37). Patients with other known interstitial lung diseases were excluded.

**Table 1:** Demographic and clinical characteristics of the study group.

**Characteristic**	**Hypersensitivity pneumonitis (*n* = 75)**	**Avian antigen exposed controls (*n* = 64)**	***p*-value**	**Non-exposed controls (*n* = 194)**	***p*-value**
Sex, male/female (%)	5/70 (8/92)	45/19 (70/30)	3.4E-16	124/70(64/36)	3.5E-17
Age (years)	44 ± 13.2	35 ± 8.8	NS	41 ± 14.5	NS
Non-smokers/smokers (%)	61/14 (81/19)	57/7 (90/10)	NS	103/91(53/47)	0.00002
FVC % predicted	56.6 ± 21.6	ND		106.5 ± 11.3^*^	
FEV1 % predicted	59.6 ± 21.7	ND		99.7 ± 12.8^*^	
FEV1/FVC%	90.7 ± 8.5	ND		79.3 ± 5.5^*^	
DL_CO_ (*n* = 43)	53.7 ± 21.4	ND		ND	
Oxygen saturation at rest (%)	85.4 ± 8.4b	ND		ND	

ND, Not done; NS, Not significant;

* Performed in 122 healthy non-exposed controls.

Paired t-test was done between HP vs. Avian antigen exposed controls and HP vs. Non-exposed controls.

Group 2 (Exposed controls) was composed of 64 healthy individuals with a history of avian antigen exposure but remained symptom-free. Some of these individuals were relatives of HP patients.

Group 3 (Non-exposed controls) was composed of 194 healthy individuals without a history of antigen exposure. Out of 194, 91 subjects were recruited randomly from the Smoking Cessation program with more than 5 years of smoking history and normal pulmonary function tests (smoker controls), and the other 103 unrelated healthy blood donors (non-smoker controls) were recruited randomly from the INER, as noted previously (13).

We collected blood samples of study participants after obtaining informed consent from subjects.

### Genotype Analysis

Genomic DNA was extracted from blood samples as described previously (8) and used as a template for polymerase chain reaction (PCR). A total of 17 targeted SNPs of surfactant protein genes, *SFTPA1, SFTPA2, SFTPB, SFTPC*, and *SFTPD*, were selected for the current study. The 17 SNPs include 5 SNPs from *SFTPA1*: rs1059047, rs1136450, rs1136451, rs1059057, and rs4253527; 4 SNPs from *SFTPA2*: rs1059046, rs17886395, rs1965707, and 1965708; 4 SNPs from *SFTPB*: rs2077079, rs3024798, rs1130866, and rs7316; 2 SNPs from *SFTPC*: rs4715 and rs1124; and 2 SNPs from *SFTPD*: rs721917 and rs2243639. The PCR-restriction fragment length polymorphism method was used to genotype the *SFTPA1, SFTPA2, SFTPD* (8, 10), *SFTPB* (10, 38), and *SFTPC* (13) gene polymorphisms, as described elsewhere in detail (8, 16). The PCR primer sequences used are reported in Supplementary Table 1. To avoid genotyping bias, each sample was given a sequential laboratory number without identifiers in the order they were received and all samples were genotyped together in a blinded fashion with those assigning genotype unaware of the group of subjects.

### Statistical Analysis

To determine the frequency of each SNP, we used the Chi-squared test, or Fisher's exact test when the expected frequency was too small (<5) and compared the allele distribution between the case and two control groups (avian antigen exposed and non-exposed). Dummy variables for each allele or SP-A genotype were created and applied for univariate logistic regression analysis, assuming no dose-effect for the alleles (13, 21). The selected markers, which were significantly associated with HP in unadjusted univariate analysis (*p*-value < 0.1), were passed to the multivariate logistic regression analysis after adjusting for smoking status and sex. Variable selection was performed using the backward elimination method with staying significance level less than 0.05.

To detect the effects of SNP-SNP interactions, we used Wang et al.'s case-control approach to study associations of SP genes polymorphisms with HP in cases vs. exposed controls and cases vs. non-exposed controls (39). Compared to traditional logistic methods, this approach is more efficient (40) in the detection of different genetic effects. It decomposes the overall genetic effect of each SNP into different components: the additive (a), recessive (r), and dominant (d) effect in a single SNP model. For two- and three-SNP models, this approach can also detect pairwise and triad-wise (high-order) epistasis, as described previously (41). If a statistically significant difference between the groups was observed, the *p*-value was corrected for variables such as sex and smoking status. The false discovery rate was controlled at 5% to account for multiple comparisons using the Benjamini- Hochberg method (42, 43). We used the Cochran's and Mantel–Haenszel test to adjust for variables (sex and smoking status) and calculated odds ratios (OR) with 95% of confidence interval (95% CI) (44). All possible SNP-SNP interactions were tested for single-, two-, and three-SNP interaction model and those with *p*-value < 0.05 are reported.

SP-A1 and SP-A2 haplotypes were assigned as described (8) and the frequency analysis was done similar to that of SNP frequencies. For haplotype estimation, we used the two-SNP haplotype estimation model to study association of haplotypes with HP patients (45). The effect of haplotype was studied in a similar way to that of the SNP-SNP interaction model in a case-control setting.

## Results

### Clinical Characteristics of HP Study Group

Table 1 shows the baseline characteristics of the HP patients, and exposed and non-exposed controls. Out of seventy-five HP patients, 92% (*n* = 70) were females compared to 30% (*n* = 19) of 64 exposed controls, and 36% (*n* = 70) of the 194 non-exposed controls (*p* < 0.00001). Only 19% (*n* = 14) of HP patients and 10% (*n* = 10) of exposed controls (*p* = NS) were current or former smokers compared to 47% (*n* = 91) of non-exposed controls (*p* = 0.00002). All HP patients demonstrated a significant reduction in FVC% and FEV1% on pulmonary function tests and hypoxemia at rest (Table 1).

### Association of SP SNP Allele With HP in Univariate and Multivariate Analysis

The observed frequency distribution of the majority of SNPs did not deviate from Hardy-Weinberg equilibrium (data not shown). The univariate and multivariate logistic regression analysis was performed to study difference in frequencies of marker allele in HP patients compared to two control groups.

#### HP Group (n = 75) vs. Avian Antigen Exposed Controls (n = 64)

Marker allele that showed significant differences (*p* < 0.1) in the univariate analysis (Table 2) were included in the multivariate analysis. After multiple logistic regression analysis, the marker allele rs7316_G of the *SFTPB* is associated with increased risk for HP, *p* = 0.03, OR (95% CI) = 4.6 (1.3–22.0), whereas, male sex appears to be associated with decreased risk for HP, *p* < 0.01, OR = 0.02 (0.01–0.08).

**Table 2:** Hypersensitivity Pneumonitis (HP) vs. avian antigen exposed controls (univariate analysis).

**Gene**	**Allele**	**HP**		**Avian antigen control**		**w/o adjusting for sex and smoking status**		**After adjusting for sex and smoking status**	
		***n***	**MAF (%)**	***n***	**MAF (%)**	**OR (95% CI)**	***p*-value**	**OR (95% CI)**	***p*-value**
*SFTPA2*	rs1965707_T	72	0.22	51	0.39	0.5 (0.2–1)	0.05	0.6 (0.2–1.9)	0.41
*SFTPA2*	rs1965708_A	72	0.19	52	0.33	0.5 (0.2–1.1)	0.1	0.8 (0.3–2.71)	0.76
*SFTPB*	rs7316_G	75	0.39	64	0.19	2.7 (1.2–6.0)	0.01	8.1 (2.3–39.3)	<0.01
*SFTPC*	rs1124_A	74	0.51	63	0.37	1.8 (0.9–3.7)	0.08	1.6 (0.6–4.1)	0.31

Percentage of n with at least one copy of the given allele. OR, odds ratio; CI, confidence interval; MAF, minor allele frequency.

#### HP Group (n = 75) vs. Non-exposed Controls (n = 194)

Similarly, marker alleles that showed significant differences (*p* < 0.1) in the univariate analysis (Table 3) were included in the multivariate analysis. Based on the OR, marker allele (rs1130866_T of the *SFTPB* and rs721917_T of the *SFTPD*), as well as male sex and smoking appear to be associated with decreased risk of HP (Table 4).

**Table 3:** Hypersensitivity Pneumonitis (HP) vs. non-exposed controls (univariate analysis).

**Gene**	**Allele**	**HP**		**Healthy control**		**w/o adjusting for sex and smoking status**		**After adjusting for sex and smoking status**	
		***n***	**MAF (%)**	***n***	**MAF (%)**	**OR (95% CI)**	***p*-value**	**OR (95% CI)**	***p*-value**
*SFTPA1*	rs1059047_C	72	0.49	192	0.4	1.4 (0.8–2.4)	0.21	1.8 (0.9–3.8)	0.1
*SFTPA1*	rs1136450_G	72	0.65	192	0.71	0.8 (0.4–1.4)	0.38	0.5 (0.2–1.0)	0.06
*SFTPA1*	rs1136451_G	72	0.58	192	0.52	1.3 (0.7–2.2)	0.37	2.0 (1.0–4.1)	0.07
*SFTPA1*	rs1059057_G	72	0.49	192	0.4	1.4 (0.8–2.5)	0.19	2.0 (1.0–4.2)	0.06
*SFTPA2*	rs1059046_C	72	0.78	193	0.71	1.5 (0.8–2.8)	0.24	2.1 (0.9–4.7)	0.07
*SFTPA2*	rs17886395_C	72	0.5	193	0.46	1.2 (0.7–2.0)	0.57	1.9 (0.9–4.0)	0.08
*SFTPB*	rs1130866_T	75	0.49	194	0.68	0.5 (0.3–0.8)	0.01	0.3 (0.2–0.7)	<0.01
*SFTPD*	rs721917_T	75	0.73	192	0.83	0.6 (0.3–1.1)	0.08	0.3 (0.1–0.6)	<0.01
*SFTPD*	rs2243639_A	75	0.61	191	0.71	0.6 (0.4–1.1)	0.12	0.4 (0.2–0.9)	0.02

OR, odds ratio; CI, confidence interval; MAF, minor allele frequency.

**Table 4:** Hypersensitivity Pneumonitis (HP) vs. non-exposed controls (multivariate analysis).

**Gene**	**Variable**	**OR (95% CI)**	***p*-value**
*SFTPB*	rs1130866_T	0.4 (0.2–0.9)	<0.01
*SFTPD*	rs721917_T	0.3 (0.1–0.7)	0.01
	Smoker	0.1 (0.05–0.3)	<0.01
	Male	0.02 (0.01–0.1)	<0.01

OR, odds ratio; CI, confidence interval.

### Association of SNP-SNP Interactions With HP

A particular SNP can have an additive (denoted as “a”), dominant (denoted as “d”) or recessive (not observed in our study) effect on the disease and the number that follows “a” or “d” indicates the position of the corresponding SNP. For example, a notation d1 x a2 x d3 means that SNPs in position 1 and 3 have dominant effect and SNP in position 2 has an additive effect on that particular interaction. These interactions could be intragenic, i.e., among SNPs of the same gene (shown in bold in the Tables), or intergenic, i.e., among SNPs of different genes. In general, each SNP exhibited either additive and/or dominant effect on HP in the single-, two-, and three-SNP model.

#### HP Group (n = 75) vs. Avian Antigen Exposed Controls (n = 64)

Since there was a statistically significant sex difference between the study groups, we adjusted for sex in the SNP analysis models. In the single- or two-SNP model, we did not observe significant association of SP genes SNPs with HP compared to exposed controls. Table 5 shows a total of 25 significant interactions associated with HP in the three-SNP model. Out of these 25 interactions, 16 were associated with increased risk for HP, *p* = 0.001–0.05, OR = 2.6–14.5, whereas 9 interactions were associated with lower risk for HP, *p* = 0.002–0.05, OR = 0.1–0.4. Out of those 25 interactions, 16 were with three dominant effects and 9 were with two dominant effects [d1 x a2 x d3 (*n* = 7), d1 x d2 x a3 (*n* = 2)]. We did not observe any significant interaction with additive effects only.

**Table 5:** Association of SP gene SNP interactions with HP compared to avian antigen exposed controls.

	**SNP # 1**	**Gene**	**SNP # 2**	**Gene**	**SNP # 3**	**Gene**	**Interaction**	**FDR**	***p*-value**	**OR (95% CI)**
1*	**rs1059046**	***SFTPA2***	**rs1059047**	***SFTPA1***	**rs2243639**	***SFTPD***	d1 x a2 x d3	**0.05**	** <0.01**	**5.4 (1.7–20.1)**
2*	rs1059046	*SFTPA2*	rs1130866	*SFTPB*	rs4715	*SFTPC*		0.05	<0.01	5.8 (1.8–22.4)
3*	rs1059046	*SFTPA2*	rs1130866	*SFTPB*	rs1124	*SFTPC*		0.05	<0.01	5.8 (1.8–22.4)
4*	**rs1059046**	***SFTPA2***	**rs721917**	***SFTPD***	**rs2243639**	***SFTPD***		**0.05**	** <0.01**	**5.0 (1.7–17.1)**
5	rs1059047	*SFTPA1*	rs1136450	*SFTPA1*	rs2077079	*SFTPB*		0.05	<0.01	0.1 (0.0–0.5)
6*	rs1059047	*SFTPA1*	rs1136451	*SFTPA1*	rs2077079	*SFTPB*		0.02	<0.01	14.5 (2.7–148.6)
7	rs2077079	*SFTPB*	rs1130866	*SFTPB*	rs721917	*SFTPD*		0.00	<0.01	0.1 (0.0–0.4)
8	rs1059046	*SFTPA2*	rs2077079	*SFTPB*	rs4715	*SFTPC*	d1 x d2 x a3	0.02	<0.01	0.1 (0.0–0.5)
9	rs1059046	*SFTPA2*	rs2077079	*SFTPB*	rs1124	*SFTPC*		0.02	<0.01	0.2 (0.0–0.5)
10*	**rs1059046**	***SFTPA2***	**rs17886395**	***SFTPA2***	**rs721917**	***SFTPD***	d1 x d2 x d3	**0.04**	** <0.01**	**2.6 (1.4–4.9)**
11*	**rs1059046**	***SFTPA2***	**rs17886395**	***SFTPA2***	**rs2243639**	***SFTPD***		**0.00**	** <0.01**	**3.2 (1.7–6.2)**
12*	**rs1059046**	***SFTPA2***	**rs1059047**	***SFTPA1***	**rs2243639**	***SFTPD***		**0.02**	** <0.01**	**2.9 (1.5–5.8)**
13*	rs1059046	*SFTPA2*	rs1136450	*SFTPA1*	rs2077079	*SFTPB*		0.00	<0.01	5.3 (2.3–13.1)
14*	rs1059046	*SFTPA2*	rs2077079	*SFTPB*	rs1130866	*SFTPB*		0.02	<0.01	2.6 (1.4–5.0)
15*	rs1059046	*SFTPA2*	rs3024798	*SFTPB*	rs1130866	*SFTPB*		0.02	<0.01	2.8 (1.5–5.8)
16	rs1059046	*SFTPA2*	rs3024798	*SFTPB*	rs721917	*SFTPD*		0.00	<0.01	0.3 (0.1–0.5)
17*	rs17886395	*SFTPA2*	rs1059047	*SFTPA1*	rs2077079	*SFTPB*		0.00	<0.01	5.7 (2.3–16.3)
18	rs17886395	*SFTPA2*	rs1136451	*SFTPA1*	rs1130866	*SFTPB*		0.00	<0.01	0.3 (0.1–0.5)
19*	**rs1059047**	***SFTPA1***	**rs1136451**	***SFTPA1***	**rs2243639**	***SFTPD***		**0.05**	** <0.01**	**2.6 (1.3–5.5)**
20*	rs1136450	*SFTPA1*	rs1136451	*SFTPA1*	rs2077079	*SFTPB*		0.05	<0.01	2.8 (1.4–6.0)
21*	**rs1136450**	***SFTPA1***	**rs1136451**	***SFTPA1***	**rs721917**	***SFTPD***		**0.02**	** <0.01**	**3.1 (1.5–6.7)**
22	rs1136450	*SFTPA1*	rs3024798	*SFTPB*	rs721917	*SFTPD*		0.04	<0.01	0.4 (0.2–0.7)
23*	rs2077079	*SFTPB*	rs3024798	*SFTPB*	rs2243639	*SFTPD*		0.01	<0.01	3.1 (1.6–6.4)
24	rs2077079	*SFTPB*	rs1130866	*SFTPB*	rs2243639	*SFTPD*		0.00	<0.01	0.3 (0.1–0.5)
25	rs3024798	*SFTPB*	rs1130866	*SFTPB*	rs2243639	*SFTPD*		0.02	<0.01	0.4 (0.2–0.7)

Sign “*” shows interactions that increased HP risk.

*Interactions among the hydrophilic SPs alone are shown in bold*.

*FDR, False discovery rate*.

Out of the 16 interactions associated with increased HP risk, 7 were among SNPs of the hydrophilic SPs (*SFTPA1, SFTPA2*, and *SFTPD*) alone, the rest (*n* = 9) were among SNPs of both the hydrophilic and hydrophobic (*SFTPB* and *SFTPC*) SPs. There were no interactions among SNPs of the hydrophobic SP genes alone. Of note, all but one interaction had SNPs of either the *SFTPA1* or *SFTPA2* gene. The exception was the interaction among two SNPs of *SFTPB* (rs2077079 x rs3024798) and the rs2243639 of *SFTPD, p* = 0.01, OR (95% CI) = 3.1(1.6–6.4).

All interactions (*n* = 9) associated with lower risk of HP were among SNPs of both hydrophilic and hydrophobic SPs. Moreover, SNPs of the *SFTPB* constituted the majority (~45%) of the SNPs in the significant interactions associated with lower risk of HP, whereas fewer than ~30% of the combined *SFTPA1* and *SFTPA2* SNPs in the interactions were associated with lower risk of HP.

#### HP Group (n = 75) vs. Non-exposed Controls (n = 194)

We observed a statistically significant difference of sex and smoking status between the groups, therefore for SNP analysis, we adjusted for both of these covariates. In the single SNP model, we observed that rs1136451 of the *SFTPA1* was associated with increased HP risk, *p* = 0.02, OR = 11.4 (2.3–57.9), whereas rs1136450 of *SFTPA1* and rs1130866 of *SFTPB* were associated with lower risk of HP, *p* = 0.02, OR = 0.2 (0.0–0.6) compared to non-exposed controls. Each of these SNPs exhibited an additive effect on HP risk, e.g., in rs1136451 (A/G), risk allele “G” exhibited an additive (GG>GA>AA) effect on increased HP risk rather than the recessive (GG>GA=AA) or dominant (GG=GA>AA) effect. We did not observe any significant interactions associated with HP in the two-SNP model. We observed a total of 97 interactions associated with HP in the three-SNP model. Out of the 97 interactions, 29 were associated with increased HP risk, *p* = 0.00009–0.05, OR = 1.9–13.3 (1.2–128.8), and the remaining 68 were associated with lower risk for HP, *p* = 0.0001–0.05, OR = 0.1–0.6 (0.0–0.9). Of the 97 interactions, (a) 3 interactions with additive effects (a1 x a2 x a3, no dominant effect of any SNP), (b) 8 interactions had one dominant effect (a1 x a2 x d3), (c) 51 interactions had two dominant effects [a1 x d2 x d3 (*n* = 9), d1 x a2 x d3 (*n* = 12), d1x d2 x a3 (*n* = 30)], and (d) 35 interactions had three dominant effects (d1 x d2 x d3) as shown in Supplementary Tables 2, 3.

**Figure 1:**
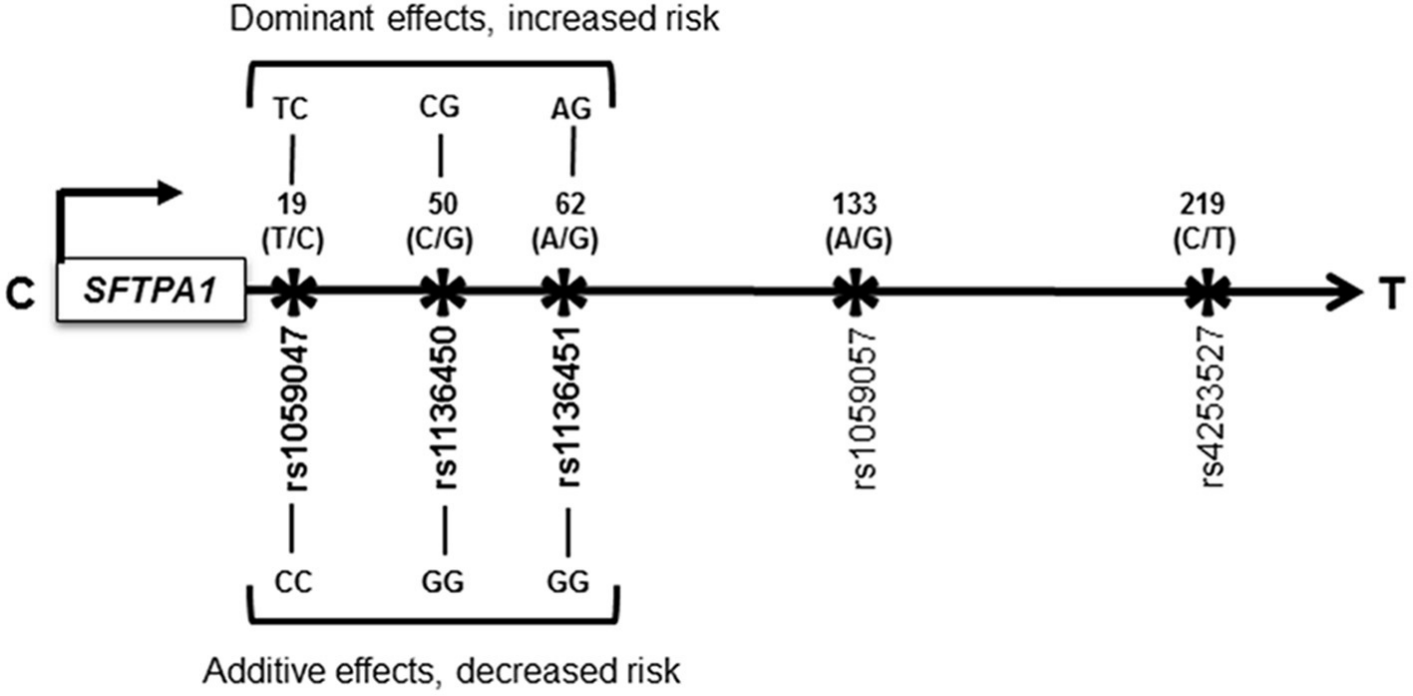
Schematic representation of the *SFTPA1* single nucleotide polymorphisms (SNPs). All the studied SNPs of the *SFTPA1* are shown. The relative location of the gene is shown from centromere (C) to telomere (T) and the arrow indicates transcriptional orientation. The number above the black arrow indicates the amino acid (AA) number of the precursor molecule and the corresponding nucleotide change shown in parenthesis. The SNP id is shown below the black arrow. The SNPs (rs1059047 x rs1136450 x rs1136451) of the *SFTPA1* involved in an intragenic interaction in the three-SNP model are shown in bold font and are associated with HP risk compared to non-exposed controls. The change for AA at the codon 19 is Val/Ala corresponding to the T/C alleles, respectively; for AA50 is Val/Leu corresponding to C/G alleles. The SNP at the codon 62 does not change the encoded amino acid. Of note, the physical location of the SNPs in this interaction is very close to each other as shown in Figure. On the basis of odds ratio, the interaction (rs1059047 x rs1136450 x rs1136451) exhibiting a dominant effect is associated with increased risk of HP (d x d x d). The dominant genotype for each SNP is shown above the black arrow. On the other hand, the same interaction is associated with decreased risk of HP when each SNP exhibited an additive effect (a x a x a). The additive genotype for each SNP is shown below the black arrow.

Of the 29 interactions associated with increased HP risk, we observed (a) one intragenic interaction among SNPs of the *SFTPA1* (rs1059047 x rs1136450 x rs1136451), where each SNP exhibited a dominant effect, *p* = 0.03, OR = 1.9 (1.2–3) (shown in Figure 1), and the remaining 28 interactions were intergenic. (b) out of the 28 intergenic interactions, seven were among SNPs of the hydrophilic SPs (*SFTPA1, SFTPA2*, and *SFTPD*) alone, and the rest were among SNPs of both hydrophobic and hydrophilic SPs. (c) all but four of the significant intergenic interactions had SNPs of either *SFTPA1* or *SFTPA2*.

Of the 68 interactions associated with lower risk of HP, we observed (a) two intragenic interactions: (i) among SNPs (rs1059047 x rs1136450 x rs1136451) of the *SFTPA1*, which were the same as the ones with increased HP risk. However, in this case, each SNP exhibited an additive effect, *p* = 0.05, OR = 0.1 (0–0.5) whereas when this interaction was associated with increased risk, each SNP exhibited a dominant effect (shown in Figure 1), and (ii) among SNPs of the *SFTPB* (rs2077079 x rs3024798 x rs1130866 exhibiting dominant x dominant x additive effect, respectively), *p* = 0.001, OR = 0.2 (0.1–0.5). (b) the remaining 66 intergenic interactions included; (i) 12 interactions among SNPs of the hydrophilic SPs alone, (ii) 3 interactions among SNPs of the hydrophobic SPs alone, and (iii) the rest (*n* = 51) were among SNPs of both hydrophobic and hydrophilic SPs.

#### Interactions That Are Common With HP in the Three-SNP Model

**Figure 2:**
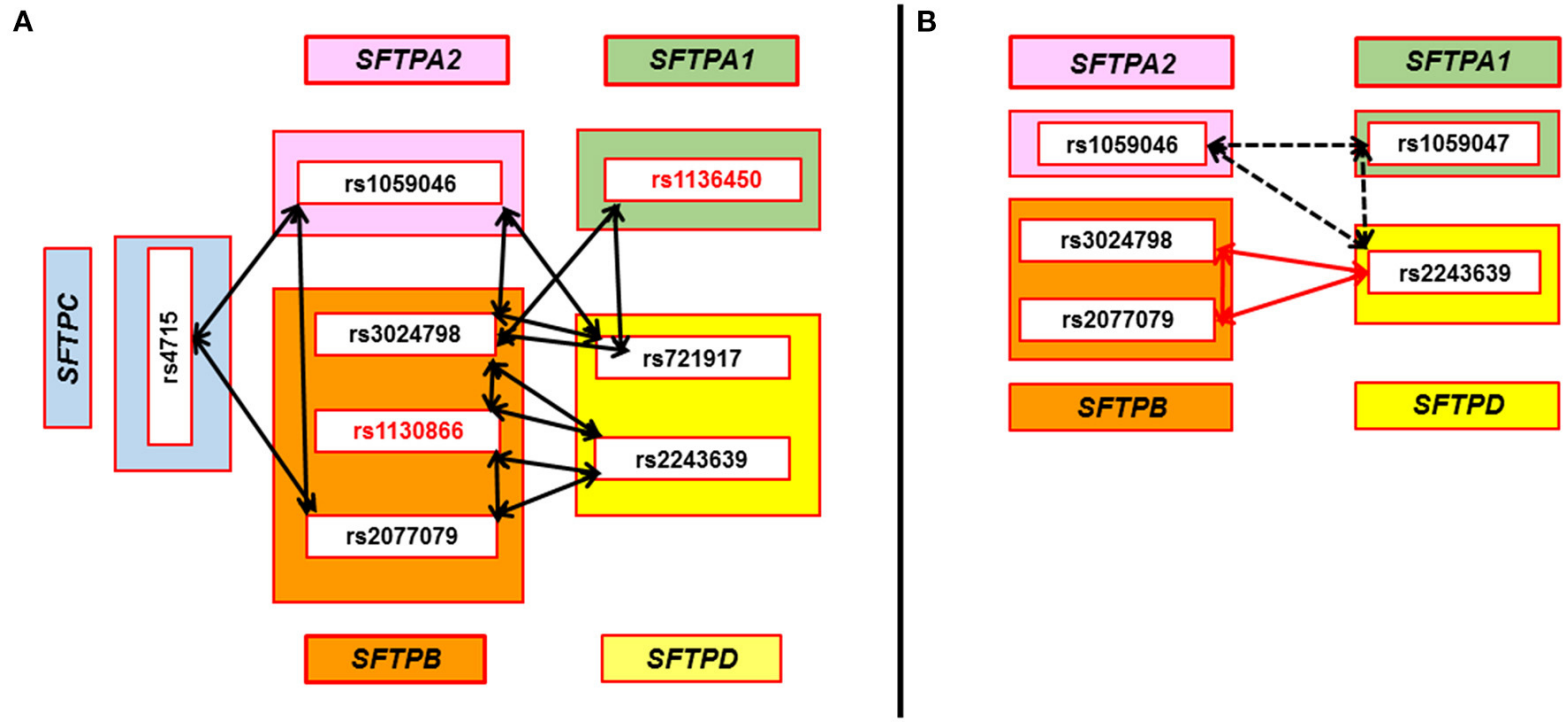
SNP-SNP interactions associated with HP compared to avian antigen exposed and non-exposed controls in the three-SNP model. Double head arrows represent common SNP-SNP interactions associated with HP and found to be significant in two sets of comparisons (HP vs. avian antigen exposed controls and HP vs. non-antigen exposed controls) in the three-SNP model. The SNPs of surfactant protein genes *SFTPA1, SFTPA2, SFTPB, SFTPC*, and *SFTPD* encoding SP-A1, SP-A2, SP-B, SP-C, and SP-D, respectively, are depicted within the green, pink, orange, blue, and yellow rectangles, respectively. **(A)** shows five significant interactions that were associated with lower risk of HP compared to antigen exposed and non-exposed controls. All these interactions were among SNPs of both hydrophobic and hydrophilic SPs and involve at least one SNP of *SFTPB* (*n* = 5) that interacts with SNPs of *SFTPD* (*n* = 4), *SFTPA1* (*n* = 1), *SFTPA2* (*n* = 2), and *SFTPC* (*n* = 1), *p* = 0.002–0.04, OR = 0.4–0.5 (0.3–0.8). Two cases of the three-SNP model involved two *SFTPB* SNPs interacting (rs3024798 and rs1130866, or rs1130866 and rs2077079) with the same *SFTPD* SNP (rs2243639). SNPs associated with lower risk of HP compared to non-exposed controls in the single-SNP model are shown in red bold font (rs1130866 of *SFTPB* and rs1136450 of *SFTPA1*). **(B)** shows two significant interactions associated with increased risk of HP compared to antigen exposed controls. One of the interactions, shown by the black dash double head arrow, is among SNPs of the hydrophilic SPs alone and was associated with increased risk of HP compared to antigen exposed and non-exposed controls, *p* = 0.009–0.05, OR = 3.5–5.4 (1.7–20.1). However, the other interaction, shown by red double head arrow among SNPs of the *SFTPB* and *SFTPD*, was associated with increased risk of HP compared to antigen exposed controls [*p* = 0.008, OR = 3.1 (1.6–6.4)]. Of interest, the same interaction was associated with lower risk of HP compared to non-exposed controls [*p* = 0.002, OR = 0.4 (0.3–0.7)].

We observed seven interactions associated with HP that were found to be in common in two separate comparisons, where cases were compared to either antigen exposed or to non-exposed controls (Figure 2). Out of the seven interactions: (a) five interactions were associated with lower risk of HP compared to antigen exposed and non-exposed controls (Panel A) and these interactions were among SNPs of both hydrophilic and hydrophobic SPs, *p* = 0.002–0.04, OR = 0.4–0.5 (0.3–0.8); (b) one was associated with increased risk of HP (Panel B – black dotted arrows) and this interaction was among SNPs of the hydrophilic SPs (rs1059046 x rs1059047 x rs2243639, *SFTPA2* x *SFTPA1* x *SFTPD*, d1 x a2 x d3), *p* = 0.009–0.05, OR = 3.5–5.4 (1.7–20.1); (c) In Panel B, one (red arrows) interaction (rs2077079 x rs3024798 x rs2243639, *SFTPB* x *SFTPB* x *SFTPD*, d1 x d2 x d3) was associated with increased risk of HP [*p* = 0.008, OR = 3.1 (1.6–6.4)] compared to antigen exposed controls, however, the same interaction was associated with lower risk of HP [*p* = 0.002, OR = 0.4 (0.3–0.7)] when compared to non-exposed controls.

### Association of Haplotypes With HP

The univariate analysis (Supplementary Table 4) showed that the frequency of several haplotypes of the *SFTPA1* and *SFTPA2* differed between HP patients vs. each of the two control groups (*p* < 0.1). In the multivariate analysis, the 1A^3^ of the *SFTPA2* and male sex were associated with decreased risk of HP compared to avian antigen control, *p* < 0.05, OR = 0.1 (1A^3^) and OR = 0.03 (male), whereas smoking and male sex, but none of the *SFTPA1* and *SFTPA2* haplotypes, appeared to decrease risk of HP compared to non-exposed healthy control, *p* < 0.01, OR = 0.13 (smoking) and OR = 0.03 (male).

**Figure 3:**
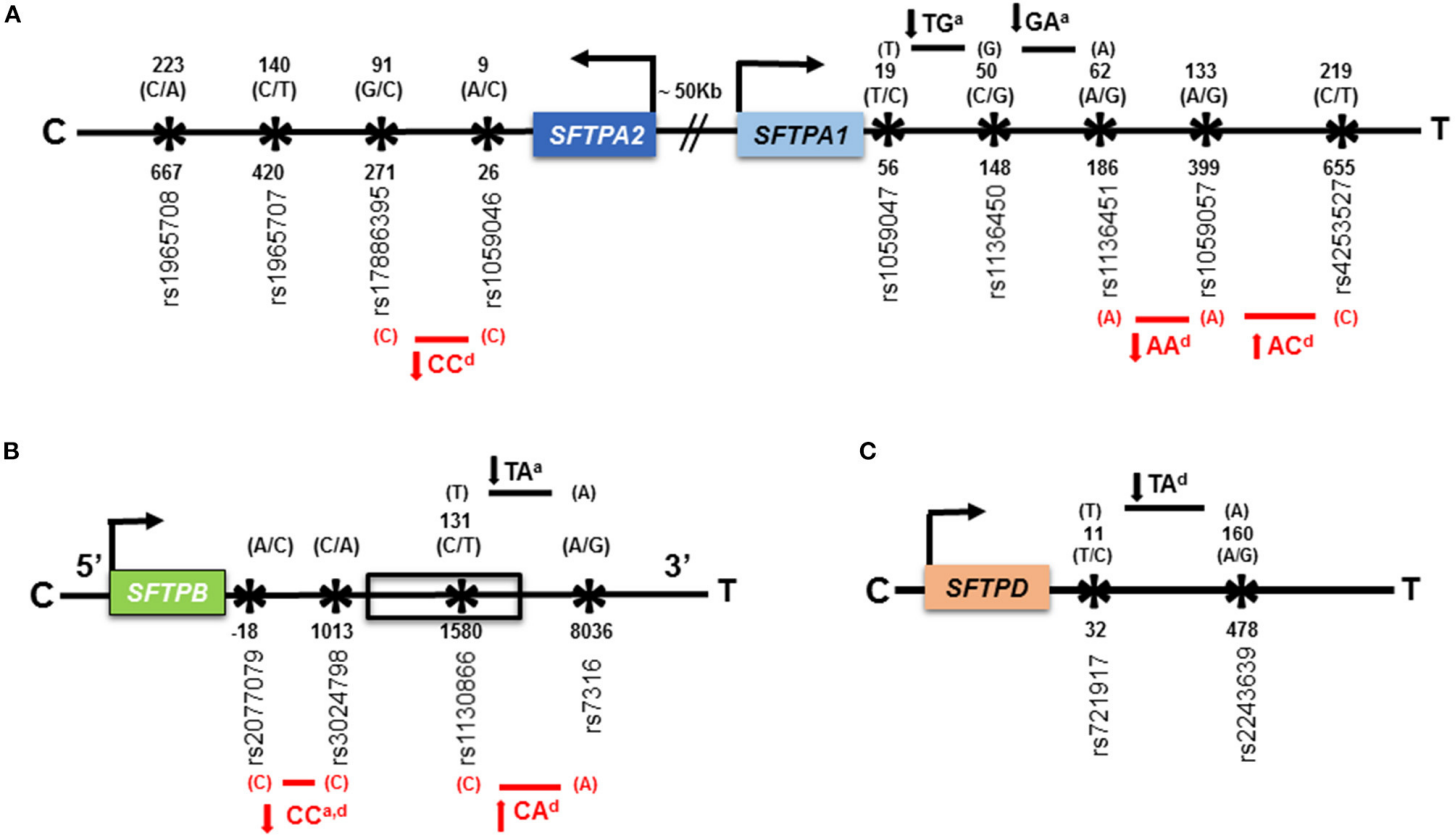
Association of haplotypes of the *SFTPA1, SFTPA2, SFTPB, and SFTPD* genes with HP compared to avian antigen exposed and non-exposed controls in the two-SNP haplotype estimation model. **(A)** is a schematic representation of SNPs of the *SFTPA1* and *SFTPA2* genes. The distance between the genes is ~50 kb shown with sign “//.” The studied SNPs are located within exons. **(B)** is a schematic representation of the *SFTPB* gene shown in 5' to 3' UTR direction. Only rs1130866 of the *SFTPB* shown in box, is located within an exon and the corresponding amino acid (AA) is shown above that. The *SFTPB* (1) rs2077079 is located 10 nucleotides downstream of TATAA box, in the 5′ regulatory region; (2) rs3024798 is located in the intron; and (3) rs7316 is located in the 3′UTR. Thus, no AA is shown for these SNPs. **(C)** is a schematic representation of SNPs of the *SFTPD* gene. The studied SNPs are located within exons. The gene direction is shown from centromere (C) to telomere (T) and the arrow above the color box indicates transcriptional orientation. The number above the black arrow indicates the AA number and the corresponding nucleotide change for that particular SNP is shown in parenthesis. The numbering of AAs in *SFTPA2, SFTPA1*, and *SFTPB* is based on the sequence of the precursor molecule, whereas, it is based on the mature protein (i.e., minus the signal peptide) in *SFTPD*. The numbers below the black arrow indicate the nucleotide number and the corresponding SNP ids. The transmitted haplotypes and nucleotide changes are shown in bold. The haplotypes associated with HP risk compared to avian antigen exposed and non-exposed controls are shown in red font with red line and black font with black line, respectively. The direction of the solid arrows besides haplotypes indicates increased or decreased HP risk compared to control groups. The superscript “a” and “d” after a given haplotype indicates additive and dominant effect, respectively, of that particular haplotype.

Using the two-SNP haplotype model, Figure 3 shows associations of haplotypes with HP compared to avian antigen exposed and non-exposed controls. Compared to avian antigen exposed controls, six haplotypes of the *SFTPA1, SFTPA2*, and *SFTPB* were associated with HP. Of the six haplotypes, four were associated with decreased risk of HP (OR = 0.02–0.3) and the remaining were associated with increased risk of HP (OR = 4.26–13.19), Supplementary Table 5. As shown in Figure 3, the haplotype “CC” of the *SFTPB* (rs2077079 (A/C) x rs3024798 (C/A)) is associated with decreased risk of HP and exhibited an additive, OR = 0.02 (0.002–0.2) as well as a dominant effect, OR = 0.12 (0.07–0.39). In this example, each parent can transmit the risk haplotype in four forms: AC, AA, CC, and CA. The additive effect of the risk haplotype “CC” means that the presence of two copies of “CC/CC” decreases the risk of HP compared to the combination of two copies of any non-risk haplotypes (AC/AC, AA/AA, and CA/CA). Whereas the dominant effect of the same haplotype “CC” means that the presence of the risk haplotype “CC” in combination with any other haplotype (AC, AA, and CA) decreases the risk of HP compared to the presence of two copies of CC or any other combination of the non-risk haplotypes (AC/AC, AC/AA, AC/CA, AA/AA, AA/CA, and CA/CA). The dominant effect of a risk haplotype may have a non-linear interaction effect in which the combination of the risk haplotype “CC” with other non-risk haplotype (AC, AA, and CA) may produce a larger effect on the disease risk than the sum of two copies of the risk haplotype “CC.” This complexity (non-linear) of interactions is our challenge for the study of human disease. Unexpected and not readily understood phenomena may occur, and this finding is one of the examples of this phenomenon. In other words, 1 + 1 > 2 in this risk haplotype. The haplotypes “CC” of the *SFTPA2* (rs1059046 x rs17886395), OR = 0.07 (0.02–0.26) and “AA” of the *SFTPA1* (rs1136451 x rs1059057), OR = 0.30 (0.12–0.78), exhibited a dominant effect on decreased HP risk. The haplotypes “AC” of the *SFTPA1* (rs1059057 x rs4253527), OR = 13.19 (4.44–39.17) and “CA” of the *SFTPB* (rs1130866 x rs7316), OR = 13.19 (4.44–39.17), exhibited a dominant effect on increased HP risk (Figure 3, red arrows).

Compared to non-exposed controls, all four significant susceptibility haplotypes of the *SFTPA1, SFTPB*, and *SFTPD* were associated with decreased risk of HP, OR = 0.15–0.35, Supplementary Table 6 and Figure 3 (black arrows). The haplotypes “TG” and “GA” of the *SFTPA1*, rs1059047 x rs1136450 and rs1136450 x rs1136451, respectively, exhibited an additive effect, OR = 0.15 (0.04–0.54). The haplotype “TA” of the *SFTPB* (rs1130866 x rs7316) exhibited an additive effect, OR = 0.17 (0.05–0.64), whereas, the haplotype “TA” of the *SFTPD* (rs721917 x rs2243639) exhibited a dominant effect, OR = 0.35 (0.18–0.66) on decreasing the risk of HP.

## Discussion

Hypersensitivity Pneumonitis is an interstitial lung disease caused by an abnormal immune response to antigen exposure and most likely due to complex interactions between environmental and genetic factors (1, 2). Surfactant proteins play an important role in normal lung function as well as innate immunity and host defense (6, 46), and alterations in their function is central to several pulmonary diseases. Several genetic variants have been identified for *SFTPA1, SFTPA2, SFTPB, SFTPC*, and *SFTPD* that are associated with pulmonary diseases (12, 47). Taking advantage of the homogeneity of the Mexican population, in the current study, we tested the hypothesis that SP genetic variants are associated with susceptibility of HP. Our results showed that (a) in the multivariate analysis, the rs7316_G of the *SFTPB* and male sex were associated with increased and decreased risk of HP, respectively, compared to antigen exposed controls, whereas the rs1130866_T of the *SFTPB*, rs721917_T of the *SFTPD*, male sex, and smoking were associated with decreased risk of HP compared to non-exposed controls; (b) in the single-SNP model, the rs1136451 of *SFTPA1* was associated with increased HP risk whereas the rs1136450 of the *SFTPA1* and the rs1130866 of the *SFTPB*, each associated with lower risk and exhibited an additive effect on HP compared to non-exposed controls; (c) in the three-SNP model, when HP patients were compared to antigen exposed and non-exposed controls, the majority of SNP-SNP interactions associated with increased risk of HP involved SNPs of the hydrophilic SPs alone, whereas, the majority of the interactions with hydrophobic SPs were associated with lower risk of HP; (d) based on OR, the 1A^3^ and “CC” haplotypes of the *SFTPA2*, each associated with decreased risk and certain haplotypes of the *SFTPA1* and *SFTPB* were associated with increased or decreased risk to HP compared to antigen exposed controls, whereas, certain haplotypes of *SFTPA1, SFTPB*, and *SFTPD*, each associated with decreased risk of HP compared to non-exposed controls. Thus, the SP genetic marker alleles, SNPs and haplotypes, either alone and/or via their interactions may contribute to the development of HP. These results also indicate that the use of multiple markers may better predict the risk of disease and be used as diagnostic and/or prognostic markers of HP.

For the current study, we used two statistical approaches: (1) a traditional logistic regression analysis, and (2) a newer SNP-SNP interaction models in case-control settings. Moreover, to avoid overestimation of an association of SNP and SNP-SNP interactions for HP patients and also to account for the heterogeneity secondary to genetic differences and environmental factors that could affect the case-control study, we used two distinct controls: (1) asymptomatic antigen exposed and (2) non-exposed healthy controls. The antigen exposed controls who did not develop disease can be classified as a resistant population and non-exposed controls represent the general population where the history of exposure is not known. Of note, 30–40% of confirmed HP patients do not have a history of antigen exposure (1) making this study clinically useful.

The regression analysis revealed a protective association of male sex and smoking with HP, although the duration and/or amount of smoking was unknown. Previous human and animal studies have shown a protective role of smoking in HP under similar antigen risk exposure (48, 49). Smoking has a paradoxical role in HP in the sense that HP develops more frequently in non-smokers than in smokers but when HP occurs in smokers, the outcome is poor (50). Similar to our study, a higher incidence of HP has been reported in females in a recent epidemiological study (49). Thus, modifiable and non-modifiable environmental factors may change the susceptibility of complex diseases. Adjusting for these significant variables and the use of two different statistical approaches could provide confidence in the observations made.

The rs7316_G of the *SFTPB* is shown to associate with increase HP risk compared to antigen exposed controls only in the multivariate regression analysis. Although the rs7316 is located in the 3′ untranslated region that frequently acts as a regulatory region affecting mRNA stability, the functional significance of this variant is unclear (51). In the single-SNP model and the univariate analysis, we found three SNPS, two of the *SFTPA1* and one of the *SFTPB*, each associated with HP compared to non-exposed controls. The rs1136451_G of the *SFTPA1* associated with increased risk of HP. This SNP at codon 62 of SP-A1 does not result in an amino acid change (proline). Moreover, the C allele of this SNP is previously shown to associate with increased risk of COPD (14) and decreased risk of TB (21) in Mexicans. Of note, the G allele of the same SNP was found to associate with decreased risk of COPD in a Chinese population (52). Though the biological effect of the variant is not known, collectively these data indicate that this variant is differentially associated with pulmonary diseases in various ethnic groups. Moreover, we observed that the rs1136450_G allele of the *SFTPA1* is associated with decreased risk of HP compared to general controls. This SNP is known to change the encoded amino acid Leucine to Valine at codon 50, but its effect on structure, function and/or stability of the SP-A is not known. Though there are some similarities in the pathogenesis of the two types of interstitial lung diseases (IPF and HP), the C allele of rs1136450 is shown to associate with six-times higher odds of developing IPF in the Mexican population compared to general controls (13), but the same allele is associated with a decreased risk of HP. Whether this along with other markers could be used to differentiate between interstitial lung diseases in Mexicans remains to be determined. A previous study has shown a 3-fold increase in SP-A in BAL of HP patients (34), however, the question remains whether increased SP-A causes or contributes to HP or it is the result of the disease process. The level of SP-A1 and SP-A2 differs among individual as a function of age and lung health status (e.g., heathy vs. cystic fibrosis, culture positive vs. culture negative), as shown by differences in the protein ration of SP-A1 to total SP-A in human BAL samples (53). In addition, several studies have shown that SPs levels are influence by age, health, smoking status, lung health disease as well as by genetic factors (54–56), however, very few studies have correlated genetic polymorphisms with serum levels (57). For the current study, we did not measure the level of SPs in BAL; therefore, the effect of SP SNPs on surfactant protein concentration is unknown in healthy and/or in HP subjects. The N-terminal segment, the collagen-like region and the neck domain but not the carbohydrate recognition domain participate in SP-A oligomerization (6). Of interest, all the significant SNPs of the *SFTPA1* and *SFTPA2* associated with HP are located within regions that participate in oligomerization, whereas SNPs (rs1965707, rs1965708, and rs4253527) located in the carbohydrate recognition domain are not associated with HP. Currently it is unknown if and how any of the significant SNPs alone or in combination contribute to SP-A oligomerization.

The rs1130866_T allele of the *SFTPB* was also associated with lower risk of HP. This SNP is a missense mutation that changes the encoded amino acid Threonine to Isoleucine and eliminates an N-linked glycosylation site (58). The T allele of rs1130866 is protective against neonatal RDS (20) and systemic sclerosis associated interstitial lung disease in a Japanese population (59). On the other hand, the C allele that has the N-linked glycosylation site is associated with increased risk of COPD (15), acute respiratory distress syndrome (10), and IPF in Mexicans (13). The presence of N-linked glycosylation may interfere with SP-B processing and protein folding in disease conditions, hence the T allele, without the N-linked glycosylation somehow protects against HP. This has been shown in a transgenic mouse model of pneumonia and sepsis, where the C allele of rs1130866 of human SP-B resulted in a decreased number of lamellar bodies, SP-B concentration, and increase surface tension compared to the T allele of rs1130866 and compared to the wild type mice (60).

In summary, the fact that the marker alleles (rs1130866_T, rs1136450_G, and rs1136451_G) identified by logistic regression analysis and SNPs identified (see below) by the single-SNP model (i.e., the additive effect of the T, G, and G alleles of the rs1130866, rs1136450, and rs1136451, respectively) are identical, based on the calculated OR (regardless of the statistical approaches used), associate with HP provide confidence that these associations are true rather than spurious.

It is known that a genetic variant in the presence of another variant can alter the susceptibility of an individual to certain diseases (61). By studying SNP-SNP interactions, we can better understand the role of genetics in complex diseases such as HP. It is possible that networks of additive and/or epistatic interactions among surfactant protein genetic variants may alter functional capabilities of certain SPs, more importantly, alveolar integrity and/or host defense at the cellular, molecular or tissue level (61). In the three-SNP model, we observed 96 interactions (HP vs. non-exposed) compared to 25 interactions (HP vs. avian antigen exposed), with only 7 interactions being in common between them. The observed difference in the number of interactions could be due to the difference in the patient population, statistical approach and/or sample size. For the current study, we enrolled HP patients, exposed and non-exposed controls from a homogeneous Mexican population and used similar statistical approach to compare HP patients with two different control groups. Therefore, the observed difference in the number of interactions is likely due to the sample size difference.

We observed 44 interactions associated with avian-antigen controls compared to non-exposed controls in a three-SNP model after adjusting for smoking status (data not shown). This indicates that although the demographics of the two control groups (antigen-exposed and non-exposed) appear identical except for the smoking status (Table 1), genetically the two control groups differ. This difference between the two control groups may be in part due to the fact that 25% of the antigen exposed controls were relatives to the cases or other. However, despite genetic differences between the two control groups, we observed seven interactions associated with HP that were found to be in common in the two separate comparisons, where HP cases were compared to either antigen exposed or to non-exposed controls (Figure 2). The association of these interactions with HP is likely robust given the two different controls and considering the baseline genetic differences between the two control groups and should be investigated further in the future.

The complex three SNP model identified various significant interactions among the hydrophilic and hydrophobic SPs associated with HP after adjusting for sex and smoking status of study participants. Interestingly, we noted that one of the common interactions, involving the *SFTPB* (rs3024798 and rs2077079) and the rs2243639 of the *SFTPD*, was associated with increased risk of HP compared to the homogeneous antigen exposed control group, however, the same interaction was associated with decreased risk of HP compared to the heterogeneous non-exposed control group, in which the history of exposure and living conditions were unknown. Heterogeneity due to differences in genetic background and/or environmental conditions is probably one of the sources of apparent discrepancies in case-control study results. Therefore, the differences indicate that caution should be exercised in the definition of cases and control groups and that findings should be interpreted within the context of experimental design.

The intragenic interaction (rs1059047 x rs1136450 x rs1136451) of the *SFTPA1* is associated with increased or decreased risk of HP depending on dominant or additive effects of each SNP, respectively, in the three-SNP model compared to non-exposed controls (Figure 1). Although the study population remained the same, the susceptibility to disease changes based on the effect the particular SNP exhibits in a particular interaction. It is possible that either too much or too little of a gene product and their interactions could lead to either over or under function, of genes in a disease state (62). Of note, the concentration and biochemical properties of surfactant proteins are altered in HP patients compared to controls (32–34). This may explain the change in HP risk based on the effect of a particular SNP in that interaction. Moreover, the majority of previous associations studies have assumed only additive effects of SNPs, without considering non-additive effects (63). By studying non-additive effects (i.e., dominant or recessive) of SNPs and their interactions in the present study, we identified associations that could change the disease risk as shown in this *SFTPA1* intragenic SNP interaction. Conversely, the same *SFTPA1* intragenic SNP interaction (rs1059047 x rs1136450 x rs1136451) with dominant effects of each SNP was associated with decreased risk of pediatric acute respiratory failure (41). The clinical outcome of a quantitative or qualitative imbalance of a given gene product in a given microenvironment, may differ among individuals. The fact that enrolled subjects in that study (41) were predominantly white children aged less than 2 years and admitted with viral infections, the contrasting effect of the same interaction on disease risk is not surprising.

Interactions of the *SFTPB* and *SFTPD* SNPs had a variable susceptibility to HP (Figure 2). For example, the rs1130866 of the *SFTPB* is associated with lower risk of HP in the single-SNP model. Interactions of that particular SNP with other *SFTPB* SNPs (rs3024798 and rs2077079); and with the rs2243639 of the *SFTPD* associated with a decreased risk of HP. These findings likely highlight the protective role of rs1130866 of the *SFTPB* in HP. However, the interaction of two other SNPs of the *SFTPB* (rs3024798 and rs2077079) with the rs2243639 of the *SFTPD* was associated with increased risk of HP. In addition, the rs1136451 of the *SFTPA1* is associated with increased risk of HP in the single-SNP model but interactions of the same SNP with other hydrophobic SPs (*SFTPB* and *SFTPC*) SNPs were associated with decreased risk of HP in the three-SNP model. As shown in the current study, a given SNP could change the susceptibility of a disease depending on its interactions with other SNPs, and this highlights the importance of studying SNP-SNP interactions rather than a single SNP association to fully understand the role of genetics in complex diseases such as HP.

In general, the majority of interactions associated with increased HP risk involved SNPs of either the *SFTPA1* and/or *SFTPA2*, whereas their interactions with the hydrophobic SPs (*SFTPB* and *SFTPC*) were associated with a decreased risk of HP. The significance for the *SFTPAs* association may be due to the differential effect of *SFTPA* gene variants in lung function parameters (29), which, in the case of HP, is also altered due to antigen-induced lung inflammation. Based on these, we speculate that the presence of the hydrophilic SP genetic variants, particularly of *SFTPA1* and *SFTPA2*, in a susceptible population contribute to a dysfunction/poor functioning of the innate immune response to avian antigen exposure, alters lung function and this in turn may contribute to the pathogenesis of HP. However, interactions of these SNPs with the hydrophobic SP gene variants, particularly with *SFTPB*, may confer protection against HP. SP-B profoundly influences intracellular processing, secretion, and the pool size of surfactant (46). SP-A and SP-B have an interactive role in maintaining surface activity *in vitro*, and both are essential components of tubular myelin, an extracellular form of surfactant (64, 65). Together, these interactions among SNPs of *SFTPA* and *SFTPB* may alter the level and/or properties of SPs in HP patients that may provide protection against dysregulated inflammation. Moreover, interactions of the *SFTPA* with *SFTPB* have been previously shown to change susceptibility to neonatal RDS based on ethnic background, where certain variants increased risk of RDS in white neonates compared to black neonates (20). Nonetheless, the impact of these actual gene-gene interactions on levels and biophysical/biochemical properties of SPs need to be studied in biological experiments.

The study of haplotypes (SNPs that are inherited together) is shown to be a powerful tool to identify associations in complex diseases such as HP (66). The 1A^3^ and “CC” haplotypes of the *SFTPA2* were associated with decreased risk of HP compared to avian antigen controls. Moreover, the 1A^3^ haplotype is shown to associate with increased risk of TB in a Mexican population compared to healthy controls. Of note, the 1A^3^ differs from most other SP-A2 haplotypes at amino acid 223 (Lys instead of Gly) located within carbohydrate recognition domain that is responsible for recognizing, binding and clearing foreign materials entering into the lungs (9). Previous studies of SP-A1 and SP-A2 variants using the transgenic mouse model showed that the 1A^3^ of the *SFTPA2* was associated with better survival (28) and exhibited significantly higher lung function compared to other SP-A1 variants (29). Thus, findings of the current study, although it is an association study, are consistent with our previous animal data where a functional outcome was measured. How this haplotype alters the functional capabilities of SP-A remains to be determined, particularly in response to a potentially dysregulated inflammation and infection. It would be interesting, guided by the SNP-SNP interaction data, to generate additional SP-A cDNAs to use them to either generate stably transfected cell lines or transgenic mice for functional and regulatory studies as done with some of the common SP-A variants (65, 67). Similar to the SNP-SNP interaction model, a haplotype estimation model showed increased or decreased risk of HP based on the effect (i.e., dominant and recessive) of that particular haplotype. It is important to note that the significant haplotypes associated with HP are located very close to each other on the gene as shown in Figure 3 and biologically have a higher chance of transmitted together.

The strengths of the study includes, (1) some of the susceptibility SNPs and SNP-SNP interactions associated with HP were the same by two different statistical approaches as well as by multiple comparisons adjusting for variables; (2) use of physiologically and biologically relevant SP SNPs, which is prerequisite for the SNP-SNP interaction model to study complex diseases (39). The limitations of the present study are the moderate sample size and the homogeneous patient population. A previous simulation study of SNP-SNP interaction indicated that a sample size of at least 100 in both case and control groups is needed to detect all the possible interactions. The moderate sample size of the current study may have resulted in under-reporting of causative SNP-SNP interactions and haplotypes, since the power to detect small differences is limited by the sample size, particularly for comparison of HP patients (*n* = 75) with antigen-exposed controls (*n* = 64). Furthermore, the findings of the present study may not be generalized in heterogeneous non-Hispanic patients. These associations should be strengthened and validated by increasing the sample size and replicating the findings in other groups of heterogeneous non-Hispanic HP patients. In addition, we did not measure SP expression/level in BAL of HP patients, therefore, the impact of these SNPs on SP expression, and in turn, on HP is unknown.

In summary, this is the first study showing association of SP SNPs and haplotypes with HP using two different statistical approaches in a Mexican population. The rs1136451 of the *SFTPA1* is associated with increased risk, whereas, the rs1136450 and the rs1130866 of the *SFTPA1* and *SFTPB*, respectively, are associated with decreased risk of HP compared to non-exposed controls using logistic regression analysis and a single-SNP model. Moreover, SNPs that are significantly associated with HP in the multivariate analysis also remained significant in the three-SNP interaction model after adjusting for smoking and sex. The rs1965707, rs1965708, and rs4253527 located within the carbohydrate recognition domain of the *SFTPA1* and *SFTPA2* were not associated with HP. SNPs of the *SFTPA1* and *SFTPA2* were overrepresented in interactions associated with increased HP risk, and their interactions with SNPs of the hydrophobic SPs for the most part associated with decreased HP risk. These observations indicate that specific SP genetic variants play role in the susceptibility of Mexicans to HP. This study focuses on complex and unique interactions of the SP SNPs with HP and gives valuable information of possible functional role of surfactant proteins in innate immunity against antigens as well as in the pathogenesis of HP. This knowledge may be useful in specific marker development for diagnosis of HP.

## Data Availability Statement

The data that support the findings of this study are available from the corresponding author, Chintan K. Gandhi, upon reasonable request.

## Ethics Statement

The studies involving human participants were reviewed and approved by National Institute of Respiratory Diseases (INER). The patients/participants provided their written informed consent to participate in this study.

## Author Contributions

CG: data curation. LY, CC, CF, SZ, RW, and CG: formal analysis. JF: funding acquisition. MS, IB-R, AP, and JF: resources. RW, MS, AP, and JF: supervision and writing – review & editing. CG, SA, and JF: writing – original draft. All authors read and approved the final manuscript.

## Conflict of Interest

The authors declare that the research was conducted in the absence of any commercial or financial relationships that could be construed as a potential conflict of interest.

## Abbreviations

HP: Hypersensitivity Pneumonitis
*SFTPA1*: gene encoding SP-A1
*SFTPA2*: gene encoding SP-A2
*SFTPB*: gene encoding SP-B
*SFTPC*: gene encoding SP-C
*SFTPD*: gene encoding SP-D
SNPs: Single nucleotide polymorphisms
IPF: Interstitial pulmonary fibrosis
COPD: Chronic obstructive pulmonary disease
RDS: Respiratory distress syndrome
OR: Odds ratio.
