# Supplementary material for: SNP and Haplotype Interaction Models Reveal Association of Surfactant Protein Gene Polymorphisms With Hypersensitivity Pneumonitis of Mexican Population
Source: Front Med (Lausanne). 2021 Jan 5;7:588404. doi: 10.3389/fmed.2020.588404 (PMC7813780; doi:10.3389/fmed.2020.588404)
Supplement: Supplementary file 1 [file Data_Sheet_1.docx]

**Supplementary Table 1** **PCR primers for the SNPs**

| **Gene** | **Variation ID** | **Nucleotide** | **Primer sequence 5' - 3'** | |
| --- | --- | --- | --- | --- |
| *SFTPA1* | rs1059047 | T/C | F | TCT GCA GGG CTC CAT ATT Gc |
|  |  |  | R | CAC ACA CTG CTC TTT TCC tC |
|  | rs1136450 | C/G | F | GCT GTG CCC TCT GGC CCT Ta |
|  |  |  | R | TCC TTT GAC ACC ATC TC |
|  | rs1136451 | A/G | F | AGA GCG TGG AGA GAA GGG GcA |
|  |  |  | R | GGG TTT GTC TGA TCC CCA TC |
|  | rs1059057 | A/G | F | CAT AAT GAC AGT AGG AGA GAA GGT CTT CTC |
|  |  |  | R | ACC CTC AGT CAG GCC TAC AT |
|  | rs4253527 | C/T | F | GGA GCC TGC AGG TCG GGG AAA Atc G |
|  |  |  | R | TCA GAA CTC ACA GAT GGT CA |
| *SFTPA2* | rs1059046 | C/A | F | ACC TCA TCT TGA TGT CAG CCT CTG GTG CaG |
|  |  |  | R | AGG GCC CAG GTC TCC TCT GA |
|  | rs17886395 | C/G | F | ACC TCA TCT TGA TGT CAG CCT CTG GTG CaG |
|  |  |  | R | AGG GCC AGG GTC TCC TcT GA |
|  | rs1965707 | C/T | F | TTT TCT CTG CAG GCC CCA TGG GTg C |
|  |  |  | R | GGG TTT GTC TGA TCC CCA TC |
|  | rs1965708 | C/A | F | TCT GCA GGG CTC CAT ATT Gc |
|  |  |  | R | CAC ACA CTG CTC TTT TCC tC |
| *SFTPB* | rs2077079 | A/C | F | GTC CAG CTA TAA GGG GCC GTG |
|  |  |  | R | GTG AGT GGT GGA GCT GCC TA |
|  | rs3024798 | C/A | F | ACT CTT GTG TCC TCC ACC TTG |
|  |  |  | R | GGC ATA GGT CAT CCT GGG CA |
|  | rs1130866 | C/T | F | CTC GAA TTC ACT CGT GAA CTC CAG CAC CC |
|  |  |  | R | GTG AGC TTG CAG CCC TCT CA |
|  | rs7316 | A/G | F | CTG TGT AAT ACA ATG TCT GCA CTA |
|  |  |  | R | CTC GAA TTC TGC TGG GAT TGC AGG TGT GA |
| *SFTPC* | rs4715 | C/A | F | GCT GAT CGC CTA CAA GCC CAG |
|  |  |  | R | CTG GAA GTT GTG GAC TTT aCT A |
|  | rs1124 | G/A | F | GAT GGA ATG CTC TCT GCA GG |
|  |  |  | R | GCA CCT CGC CAC ACA GGG aG |
| *SFTPD* | rs721917 | T/C | F | CTC CTC TCT GCA CTG GTC AT |
|  |  |  | R | ACC AGG GTG CAA GCA CTG cG |
|  | rs2243639 | A/G | F | AGC GTG GAG TCC CTG GAA gC |
|  |  |  | R | AGA TTC TCT CCA TGT TCC CAG |

Lower case: mismatch to DNA sequence

**Supplementary Table 2 Association of SP gene SNPs with increased HP risk compared to non-exposed controls**

|  | **SNP # 1** | **Gene** | **SNP # 2** | **Gene** | **SNP # 3** | **Gene** | **Interaction** | **FDR** | **p value** | **OR (95% CI)** |
| --- | --- | --- | --- | --- | --- | --- | --- | --- | --- | --- |
| **Single SNP model** | | | | | | | | | | |
| 1 | rs1136451 | *SFTPA1* |  |  |  |  | Additive | 0.02 | <0.01 | 11.4 (2.3-57.9) |
| **Three-SNP model** | | | | | | | | | | |
| 1 | rs1059046 | *SFTPA2* | rs1059047 | *SFTPA1* | rs2077079 | *SFTPB* | a1 x a2 x d3 | 0.05 | <0.01 | 6.5 (1.8-30.3) |
| 2 | rs1059046 | *SFTPA2* | rs1059047 | *SFTPA1* | rs3024798 | *SFTPB* |  | 0.01 | <0.01 | 13.3 (2.8-128.8) |
| 3 | rs17886395 | *SFTPA2* | rs1136451 | *SFTPA1* | rs3024798 | *SFTPB* |  | 0.05 | <0.01 | 5.8 (1.9-21.4) |
| 4 | rs1059047 | *SFTPA1* | rs1130866 | *SFTPB* | rs2243639 | *SFTPD* | a1 x d2 x d3 | 0.02 | <0.01 | 3.2 (1.6-6.7) |
| 5 | rs1136451 | *SFTPA1* | rs2077079 | *SFTPB* | rs3024798 | *SFTPB* |  | 0.00 | <0.01 | 9.2 (3.3-29.9) |
| 6 | rs3024798 | *SFTPB* | rs721917 | *SFTPD* | rs2243639 | *SFTPD* |  | 0.02 | <0.01 | 3.4 (1.6-7.6) |
| 7 | **rs1059046** | ***SFTPA2*** | **rs1059047** | ***SFTPA1*** | **rs2243639** | ***SFTPD*** | d1 x a2 x d3 | **0.01** | **<0.01** | **3.5 (1.7-7.6)** |
| 8 | rs1130866 | *SFTPB* | rs4715 | *SFTPC* | rs721917 | *SFTPD* |  | 0.01 | <0.01 | 3.5 (1.7-7.5) |
| 9 | rs1130866 | *SFTPB* | rs1124 | *SFTPC* | rs721917 | *SFTPD* |  | 0.02 | <0.01 | 3.1 (1.5-6.3) |
| 10 | **rs1059046** | ***SFTPA2*** | **rs1136450** | ***SFTPA1*** | **rs1136451** | ***SFTPA1*** | d1 x d2 x a3 | **0.05** | **<0.01** | **3.6 (1.3-10.6)** |
| 11 | **rs1059046** | ***SFTPA2*** | **rs1136450** | ***SFTPA1*** | **rs721917** | ***SFTPD*** |  | **0.01** | **<0.01** | **3.7 (1.7-8.2)** |
| 12 | **rs17886395** | ***SFTPA2*** | **rs1059047** | ***SFTPA1*** | **rs1136451** | ***SFTPA1*** |  | **0.01** | **<0.01** | **6.0 (2.0-21.9)** |
| 13 | rs17886395 | *SFTPA2* | rs1136451 | *SFTPA1* | rs2077079 | *SFTPB* |  | 0.01 | <0.01 | 3.8 (1.7-9.0) |
| 14 | rs17886395 | *SFTPA2* | rs1136451 | *SFTPA1* | rs3024798 | *SFTPB* |  | 0.03 | <0.01 | 3.0 (1.4-6.7) |
| 15 | rs17886395 | *SFTPA2* | rs2077079 | *SFTPB* | rs2243639 | *SFTPD* |  | 0.00 | <0.01 | 4.5 (2.1-10.6) |
| 16 | **rs1136450** | ***SFTPA1*** | **rs1136451** | ***SFTPA1*** | **rs721917** | ***SFTPD*** |  | **0.03** | **<0.01** | **3.2 (1.4-7.5)** |
| 17 | **rs1136450** | ***SFTPA1*** | **rs1136451** | ***SFTPA1*** | **rs2243639** | ***SFTPD*** |  | **0.01** | **<0.01** | **4.6 (1.9-11.9)** |
| 18 | rs1136451 | *SFTPA1* | rs1130866 | *SFTPB* | rs2243639 | *SFTPD* |  | 0.04 | <0.01 | 3.1 (1.3-7.9) |
| 19 | rs2077079 | *SFTPB* | rs3024798 | *SFTPB* | rs721917 | *SFTPD* |  | 0.00 | <0.01 | 7.9 (3.2-22.1) |
| 20 | **rs1059046** | ***SFTPA2*** | **rs17886395** | ***SFTPA2*** | **rs1136451** | ***SFTPA1*** | d1 x d2 x d3 | **0.01** | **<0.01** | **2.0 (1.3-3.2)** |
| 21 | rs17886395 | *SFTPA2* | rs2077079 | *SFTPB* | rs3024798 | *SFTPB* |  | 0.00 | <0.01 | 2.2 (1.4-3.4) |
| 22 | rs17886395 | *SFTPA2* | rs2077079 | *SFTPB* | rs1130866 | *SFTPB* |  | 0.01 | <0.01 | 2.0 (1.3-3.2) |
| 23† | **rs1059047** | ***SFTPA1*** | **rs1136450** | ***SFTPA1*** | **rs1136451** | ***SFTPA1*** |  | **0.03** | **<0.01** | **1.9 (1.2-3.0)** |
| 24 | rs1059047 | *SFTPA1* | rs2077079 | *SFTPB* | rs3024798 | *SFTPB* |  | 0.01 | <0.01 | 2.1 (1.4-3.2) |
| 25 | rs1136450 | *SFTPA1* | rs2077079 | *SFTPB* | rs1130866 | *SFTPB* |  | 0.00 | <0.01 | 2.1 (1.4-3.3) |
| 26 | rs1136450 | *SFTPA1* | rs3024798 | *SFTPB* | rs1130866 | *SFTPB* |  | 0.02 | <0.01 | 1.9 (1.2-2.9) |
| 27 | rs1136450 | *SFTPA1* | rs3024798 | *SFTPB* | rs2243639 | *SFTPD* |  | 0.02 | <0.01 | 2.0 (1.3-3.1) |
| 28 | rs1136451 | *SFTPA1* | rs2077079 | *SFTPB* | rs1124 | *SFTPC* |  | 0.03 | <0.01 | 1.9 (1.2-2.9) |
| 29 | rs1136451 | *SFTPA1* | rs3024798 | *SFTPB* | rs2243639 | *SFTPD* |  | 0.04 | <0.01 | 1.8 (1.2-2.9) |

Sign “†” shows intragenic interactions among SNPs of *SFTPA1*

Intergenic interactions among the hydrophilic SPs alone are shown in bold

FDR – False discovery rate

**Supplementary Table 3 Association of SP gene SNPs with lower HP risk compared to non-exposed controls**

|  | **SNP # 1** | **Gene** | **SNP # 2** | **Gene** | **SNP # 3** | **Gene** | **Interaction** | **FDR** | **p value** | **OR (95% CI)** |
| --- | --- | --- | --- | --- | --- | --- | --- | --- | --- | --- |
| **Single SNP model** | | | | | | | | | | |
| 1 | rs1136450 | *SFTPA1* |  |  |  |  | Additive | 0.02 | <0.01 | 0.2 (0-0.6) |
| 2 | rs1130866 | *SFTPB* |  |  |  |  | Additive | 0.02 | <0.01 | 0.2 (0.1-0.5) |
| **Three-SNP model** | | | | | | | | | | |
| 1† | rs1059047 | *SFTPA1* | rs1136450 | *SFTPA1* | rs1136451 | *SFTPA1* | a1 x a2 x a3 | 0.05 | <0.01 | 0.1 (0.0-0.5) |
| 2 | **rs1059047** | ***SFTPA1*** | **rs1136450** | ***SFTPA1*** | **rs721917** | ***SFTPD*** |  | **0.03** | **<0.01** | **0.1 (0.0-0.4)** |
| 3 | **rs1136450** | ***SFTPA1*** | **rs1136451** | ***SFTPA1*** | **rs721917** | ***SFTPD*** |  | **0.04** | **<0.01** | **0.1 (0.0-0.5)** |
| 4 | rs1059046 | *SFTPA2* | rs17886395 | *SFTPA2* | rs2077079 | *SFTPB* | a1 x a2 x d3 | 0.05 | <0.01 | 0.2 (0.0-0.5) |
| 5 | rs1059046 | *SFTPA2* | rs17886395 | *SFTPA2* | rs3024798 | *SFTPB* |  | 0.01 | <0.01 | 0.1 (0.0-0.4) |
| 6 | **rs17886395** | ***SFTPA2*** | **rs1136450** | ***SFTPA1*** | **rs2243639** | ***SFTPD*** |  | **0.05** | **<0.01** | **0.1 (0.0-0.5)** |
| 7 | rs1059047 | *SFTPA1* | rs1136451 | *SFTPA1* | rs3024798 | *SFTPB* |  | 0.05 | <0.01 | 0.2 (0.1-0.6) |
| 8 | rs2077079 | *SFTPB* | rs3024798 | *SFTPB* | rs2243639 | *SFTPD* |  | 0.05 | <0.01 | 0.2 (0.1-0.6) |
| 9 | **rs1059046** | ***SFTPA2*** | **rs1136450** | ***SFTPA1*** | **rs1136451** | ***SFTPA1*** | a1 x d2 x d3 | **0.00** | **<0.01** | **0.1 (0.0-0.4)** |
| 10 | rs1059046 | *SFTPA2* | rs2077079 | *SFTPB* | rs3024798 | *SFTPB* |  | 0.02 | <0.01 | 0.3 (0.1-0.6) |
| 11 | rs1136450 | *SFTPA1* | rs2077079 | *SFTPB* | rs3024798 | *SFTPB* |  | 0.00 | <0.01 | 0.2 (0.1-0.5) |
| 12 | rs1136450 | *SFTPA1* | rs1130866 | *SFTPB* | rs721917 | *SFTPD* |  | 0.00 | <0.01 | 0.2 (0.1-0.4) |
| 13 | rs1136450 | *SFTPA1* | rs4715 | *SFTPC* | rs1124 | *SFTPC* |  | 0.00 | <0.01 | 0.1 (0.0-0.3) |
| 14* | rs1130866 | *SFTPB* | rs4715 | *SFTPC* | rs1124 | *SFTPC* |  | 0.05 | <0.01 | 0.3 (0.2-0.7) |
| 15 | **rs1059046** | ***SFTPA2*** | **rs17886395** | ***SFTPA2*** | **rs1136450** | ***SFTPA1*** | d1 x a2 x d3 | **0.03** | **<0.01** | **0.3 (0.1-0.7)** |
| 16 | rs1059046 | *SFTPA2* | rs17886395 | *SFTPA2* | rs1124 | *SFTPC* |  | 0.01 | <0.01 | 0.2 (0.1-0.5) |
| 17 | **rs1059046** | ***SFTPA2*** | **rs17886395** | ***SFTPA2*** | **rs2243639** | ***SFTPD*** |  | **0.01** | **<0.01** | **0.3 (0.1-0.6)** |
| 18 | **rs1059046** | ***SFTPA2*** | **rs1136450** | ***SFTPA1*** | **rs1136451** | ***SFTPA1*** |  | **0.00** | **<0.01** | **0.1 (0.0-0.3)** |
| 19 | rs17886395 | *SFTPA2* | rs2077079 | *SFTPB* | rs3024798 | *SFTPB* |  | 0.01 | <0.01 | 0.2 (0.0-0.5) |
| 20 | rs1059047 | *SFTPA1* | rs2077079 | *SFTPB* | rs3024798 | *SFTPB* |  | 0.01 | <0.01 | 0.1 (0.0-0.4) |
| 21 | rs1059047 | *SFTPA1* | rs1130866 | *SFTPB* | rs721917 | *SFTPD* |  | 0.03 | <0.01 | 0.3 (0.1-0.7) |
| 22 | rs1136450 | *SFTPA1* | rs1130866 | *SFTPB* | rs721917 | *SFTPD* |  | 0.02 | <0.01 | 0.3 (0.1-0.6) |
| 23 | rs1136451 | *SFTPA1* | rs2077079 | *SFTPB* | rs3024798 | *SFTPB* |  | 0.01 | <0.01 | 0.2 (0.0-0.5) |
| 24 | **rs1059046** | ***SFTPA2*** | **rs17886395** | ***SFTPA2*** | **rs1136450** | ***SFTPA1*** | d1 x d2 x a3 | **0.04** | **<0.01** | **0.3 (0.1-0.7)** |
| 25 | rs1059046 | *SFTPA2* | rs17886395 | *SFTPA2* | rs1130866 | *SFTPB* |  | 0.01 | <0.01 | 0.3 (0.1-0.6) |
| 26 | rs1059046 | *SFTPA2* | rs17886395 | *SFTPA2* | rs1124 | *SFTPC* |  | 0.04 | <0.01 | 0.4 (0.2-0.8) |
| 27 | rs1059046 | *SFTPA2* | rs1136451 | *SFTPA1* | rs1130866 | *SFTPB* |  | 0.00 | <0.01 | 0.2 (0.1-0.4) |
| 28 | rs1059046 | *SFTPA2* | rs2077079 | *SFTPB* | rs4715 | *SFTPC* |  | 0.03 | <0.01 | 0.4 (0.2-0.7) |
| 29 | rs1059046 | *SFTPA2* | rs3024798 | *SFTPB* | rs4715 | *SFTPC* |  | 0.05 | <0.01 | 0.4 (0.2-0.8) |
| 30 | rs17886395 | *SFTPA2* | rs1059047 | *SFTPA1* | rs1130866 | *SFTPB* |  | 0.01 | <0.01 | 0.3 (0.1-0.7) |
| 31 | rs17886395 | *SFTPA2* | rs1136451 | *SFTPA1* | rs1130866 | *SFTPB* |  | 0.01 | <0.01 | 0.3 (0.1-0.7) |
| 32 | rs17886395 | *SFTPA2* | rs2077079 | *SFTPB* | rs3024798 | *SFTPB* |  | 0.00 | <0.01 | 0.1 (0.0-0.4) |
| 33 | rs17886395 | *SFTPA2* | rs3024798 | *SFTPB* | rs1130866 | *SFTPB* |  | 0.03 | <0.01 | 0.3 (0.1-0.7) |
| 34 | rs17886395 | *SFTPA2* | rs4715 | *SFTPC* | rs721917 | *SFTPD* |  | 0.03 | <0.01 | 0.3 (0.1-0.7) |
| 35 | rs1059047 | *SFTPA1* | rs2077079 | *SFTPB* | rs3024798 | *SFTPB* |  | 0.00 | <0.01 | 0.1 (0.0-0.4) |
| 36 | rs1059047 | *SFTPA1* | rs2077079 | *SFTPB* | rs1130866 | *SFTPB* |  | 0.02 | <0.01 | 0.3 (0.1-0.7) |
| 37 | rs1059047 | *SFTPA1* | rs3024798 | *SFTPB* | rs1130866 | *SFTPB* |  | 0.01 | <0.01 | 0.2 (0.1-0.6) |
| 38 | rs1136450 | *SFTPA1* | rs2077079 | *SFTPB* | rs4715 | *SFTPC* |  | 0.00 | <0.01 | 0.3 (0.1-0.6) |
| 39 | rs1136450 | *SFTPA1* | rs3024798 | *SFTPB* | rs4715 | *SFTPC* |  | 0.02 | <0.01 | 0.3 (0.2-0.7) |
| 40 | rs1136451 | *SFTPA1* | rs3024798 | *SFTPB* | rs1130866 | *SFTPB* |  | 0.01 | <0.01 | 0.3 (0.1-0.6) |
| 41† | rs2077079 | *SFTPB* | rs3024798 | *SFTPB* | rs1130866 | *SFTPB* |  | 0.00 | <0.01 | 0.2 (0.1-0.5) |
| 42 | rs2077079 | *SFTPB* | rs1130866 | *SFTPB* | rs721917 | *SFTPD* |  | 0.04 | <0.01 | 0.4 (0.2-0.8) |
| 43* | rs1130866 | *SFTPB* | rs4715 | *SFTPC* | rs1124 | *SFTPC* |  | 0.04 | <0.01 | 0.3 (0.1-0.8) |
| 44 | rs1059046 | *SFTPA2* | rs1136450 | *SFTPA1* | rs1124 | *SFTPC* | d1 x d2 x d3 | 0.05 | <0.01 | 0.6 (0.4-0.9) |
| 45 | **rs1059046** | ***SFTPA2*** | **rs1136450** | ***SFTPA1*** | **rs721917** | ***SFTPD*** |  | **0.00** | **<0.01** | **0.4 (0.3-0.7)** |
| 46 | **rs1059046** | ***SFTPA2*** | **rs1136450** | ***SFTPA1*** | **rs2243639** | ***SFTPD*** |  | **0.00** | **<0.01** | **0.4 (0.3-0.6)** |
| 47 | rs1059046 | *SFTPA2* | rs1136451 | *SFTPA1* | rs4715 | *SFTPC* |  | 0.00 | <0.01 | 0.4 (0.2-0.6) |
| 48 | rs1059046 | *SFTPA2* | rs1136451 | *SFTPA1* | rs1124 | *SFTPC* |  | 0.01 | <0.01 | 0.5 (0.3-0.7) |
| 49 | **rs1059046** | ***SFTPA2*** | **rs1136451** | ***SFTPA1*** | **rs2243639** | ***SFTPD*** |  | **0.01** | **<0.01** | **0.5 (0.3-0.8)** |
| 50 | rs1059046 | *SFTPA2* | rs3024798 | *SFTPB* | rs721917 | *SFTPD* |  | 0.04 | <0.01 | 0.6 (0.4-0.9) |
| 51 | rs1059046 | *SFTPA2* | rs1130866 | *SFTPB* | rs721917 | *SFTPD* |  | 0.01 | <0.01 | 0.5 (0.3-0.7) |
| 52 | rs1059046 | *SFTPA2* | rs1130866 | *SFTPB* | rs2243639 | *SFTPD* |  | 0.00 | <0.01 | 0.3 (0.2-0.6) |
| 53 | rs1059046 | *SFTPA2* | rs1124 | *SFTPC* | rs721917 | *SFTPD* |  | 0.00 | <0.01 | 0.4 (0.3-0.6) |
| 54 | rs17886395 | *SFTPA2* | rs1136451 | *SFTPA1* | rs3024798 | *SFTPB* |  | 0.00 | <0.01 | 0.4 (0.3-0.6) |
| 55 | rs1059047 | *SFTPA1* | rs1136451 | *SFTPA1* | rs3024798 | *SFTPB* |  | 0.00 | <0.01 | 0.4 (0.3-0.7) |
| 56 | rs1136450 | *SFTPA1* | rs1136451 | *SFTPA1* | rs4715 | *SFTPC* |  | 0.00 | <0.01 | 0.4 (0.2-0.6) |
| 57 | rs1136450 | *SFTPA1* | rs1136451 | *SFTPA1* | rs1124 | *SFTPC* |  | 0.02 | <0.01 | 0.5 (0.3-0.8) |
| 58 | **rs1136450** | ***SFTPA1*** | **rs1136451** | ***SFTPA1*** | **rs2243639** | ***SFTPD*** |  | **0.01** | **<0.01** | **0.5 (0.3-0.8)** |
| 59 | rs1136450 | *SFTPA1* | rs2077079 | *SFTPB* | rs721917 | *SFTPD* |  | 0.00 | <0.01 | 0.5 (0.3-0.7) |
| 60 | rs1136450 | *SFTPA1* | rs3024798 | *SFTPB* | rs721917 | *SFTPD* |  | 0.00 | <0.01 | 0.4 (0.3-0.7) |
| 61 | rs1136450 | *SFTPA1* | rs1130866 | *SFTPB* | rs721917 | *SFTPD* |  | 0.00 | <0.01 | 0.3 (0.2-0.4) |
| 62 | rs1136450 | *SFTPA1* | rs1130866 | *SFTPB* | rs2243639 | *SFTPD* |  | 0.00 | <0.01 | 0.2 (0.1-0.3) |
| 63 | rs1136450 | *SFTPA1* | rs1124 | *SFTPC* | rs721917 | *SFTPD* |  | 0.02 | <0.01 | 0.5 (0.3-0.8) |
| 64 | rs2077079 | *SFTPB* | rs3024798 | *SFTPB* | rs2243639 | *SFTPD* |  | 0.00 | <0.01 | 0.4 (0.3-0.7) |
| 65 | rs2077079 | *SFTPB* | rs1130866 | *SFTPB* | rs2243639 | *SFTPD* |  | 0.01 | <0.01 | 0.5 (0.3-0.7) |
| 66 | rs3024798 | *SFTPB* | rs1130866 | *SFTPB* | rs721917 | *SFTPD* |  | 0.01 | <0.01 | 0.5 (0.3-0.8) |
| 67 | rs3024798 | *SFTPB* | rs1130866 | *SFTPB* | rs2243639 | *SFTPD* |  | 0.01 | <0.01 | 0.5 (0.3-0.8) |
| 68* | rs3024798 | *SFTPB* | rs4715 | *SFTPC* | rs1124 | *SFTPC* |  | 0.01 | <0.01 | 0.5 (0.3-0.7) |

Sign “†” shows intragenic interactions

Sign “*” shows intergenic interactions among the hydrophobic SPs alone

Intergenic interactions among the hydrophilic SPs alone are shown in bold

FDR – False discovery rate

**Supplementary Table 4 Association of SP-A haplotypes with HP patients compared to avian antigen exposed and non-exposed controls in univariate analysis**

| **Haplotype** | **HP** | | **Avian antigen control** | | **w/o adjusting for sex and smoking status** | | **After adjusting for sex and smoking status** | |
| --- | --- | --- | --- | --- | --- | --- | --- | --- |
|  | **n** | **MAF (%)** | **n** | **MAF (%)** | **OR (95% CI)** | **p value** | **OR (95% CI)** | **p value** |
| 1A^3^ | 72 | 0.03 | 52 | 0.08 | 0.3 (0.05 - 1.8) | 0.23 | 0.1 (0.01 - 0.7) | 0.02 |
|  | **HP** | | **Healthy control** | | **w/o adjusting for sex and smoking status** | | **After adjusting for sex and smoking status** | |
|  | **n** | **MAF (%)** | **n** | **MAF (%)** | **OR (95% CI)** | **p value** | **OR (95% CI)** | **p value** |
| 1A^5^ | 72 | 0.03 | 188 | 0.03 | 1.0 (0.1 - 5) | 0.96 | 9.4 (1.0 - 71.6) | 0.03 |
| 6A | 72 | 0.47 | 192 | 0.4 | 1.4 (0.8 - 2.4) | 0.26 | 1.9 (0.9 - 4.0) | 0.07 |
| 6A^2^ | 72 | 0.65 | 192 | 0.71 | 0.8 (0.4 - 1.4) | 0.38 | 0.5 (0.2 - 1.0) | 0.06 |

OR= odds ratio, CI= confidence interval, MAF- minor allele frequency

**Supplementary Table 5 Association of risk haplotypes with HP patients compared to avian antigen exposed controls**

| **SNP # 1** | **SNP # 2** | **Gene** | **Chr** | **Haplotype** | **Effect** | **p value** | **FDR** | **OR (95% CI)** |
| --- | --- | --- | --- | --- | --- | --- | --- | --- |
| rs2077079 | rs3024798 | *SFTPB* | 2 | CC | Additive | 0 | 0 | 0.02 (0.002-0.2) |
| rs1059046 | rs17886395 | *SFTPA2* | 10 | CC | Dominant | 0 | 0 | 0.07 (0.02-0.26) |
| rs1136451 | rs1059057 | *SFTPA1* | 10 | AA | Dominant | 0.013 | 0.015 | 0.30 (0.12-0.78) |
| rs2077079 | rs3024798 | *SFTPB* | 2 | CC | Dominant | 0 | 0 | 0.12 (0.07-0.39) |
| rs1130866 | rs7316 | *SFTPB* | 2 | CA | Dominant | 0 | 0 | 13.19 (4.44-39.17) |
| rs1059057 | rs4253527 | *SFTPA1* | 10 | AC | Dominant | 0.009 | 0.011 | 4.26 (1.48-12.24) |

Chr- Chromosome

**Supplementary Table 6 Association of risk haplotypes with HP patients compared to non-exposed controls**

| **SNP # 1** | **SNP # 2** | **Gene** | **Chr** | **Haplotype** | **Effect** | **p value** | **FDR** | **OR (95% CI)** |
| --- | --- | --- | --- | --- | --- | --- | --- | --- |
| rs1059047 | rs1136450 | *SFTPA1* | 10 | TG | Additive | <0.01 | 0.0207 | 0.15 (0.04-0.52) |
| rs1136450 | rs1136451 | *SFTPA1* | 10 | GA | Additive | <0.01 | 0.0207 | 0.15 (0.04-0.54) |
| rs1130866 | rs7316 | *SFTPB* | 2 | TA | Additive | <0.01 | 0.0484 | 0.17 (0.05-0.64) |
| rs721917 | rs2243639 | *SFTPD* | 10 | TA | Dominant | <0.01 | 0.0148 | 0.35 (0.18-0.66) |

Chr- Chromosome
